# Supplementary material for: A refinement approach in a mouse model of rehabilitation research. Analgesia strategy, reduction approach and infrared thermography in spinal cord injury
Source: PLoS One. 2019 Oct 30;14(10):e0224337. doi: 10.1371/journal.pone.0224337 (PMC6821094; doi:10.1371/journal.pone.0224337)
Supplement: S1 File — (PDF) [file pone.0224337.s001.pdf]

ID	Day	BMS	Twitching	Grooming	Digging	Ear	Temp30
		Score	*n°event/10 min*	*n°event/10 min*	*n°event/10 min*	*Score*	
13	Baseline	NA	0	8	11	0	30,24
14	Baseline	NA	0	7	2	0	30,88
15	Baseline	NA	0	3	1	0	30,58
16	Baseline	NA	0	6	3	0	31,25
17	Baseline	NA	0	5	2	0	30,7
18	Baseline	NA	0	9	0	0	30,15
19	Baseline	NA	0	11	0	0	30,59
20	Baseline	NA	0	7	0	0	29,36
21	Baseline	NA	0	1	2	0	30,9
22	Baseline	NA	0	2	0	0	31,44
23	Baseline	NA	0	2	1	0	31,06
24	Baseline	NA	0	2	0	0	30,98
13	Day 0	0	10	0	0	2	31,84
14	Day 0	0	6	5	0	1,5	32,6
15	Day 0	0	18	6	0	0,5	31,82
16	Day 0	0	5	6	0	1	31,93
17	Day 0	0	19	3	2	1	32,22
18	Day 0	0	16	16	0	2	29,25
19	Day 0	0	10	1	0	1	30,61
20	Day 0	0	7	0	0	1,5	29,2
21	Day 0	0	4	1	0	2	30,48
22	Day 0	0	5	0	0	1	30,01
23	Day 0	0	9	3	0	1,5	31,3
24	Day 0	0	5	1	0	1	31,1
13	Day 1	0	1	0	0	2	33,93
14	Day 1	0	2	5	0	1	34,26
15	Day 1	0	0	5	5	2	33,7
16	Day 1	0	1	4	6	1,5	33,57
17	Day 1	0	3	4	6	1,5	32,59
18	Day 1	0	1	3	6	2	31,72
19	Day 1	0	1	0	4	1	32,56
20	Day 1	0	0	2	3	2	32,08
21	Day 1	0	2	9	0	2	32,28
22	Day 1	0	3	0	0	0,5	32,07
23	Day 1	0	0	0	2	0,5	31,35
24	Day 1	0	0	11	0	0,5	33,93
13	Day 7	1	0	0	5	0	36,31
14	Day 7	0	0	6	6	0,5	36,67
15	Day 7	0	0	4	9	0	35,64
16	Day 7	1	0	3	11	0,5	36,27
17	Day 7	1	0	3	10	0	36,43
18	Day 7	0	0	3	5	0	35,76
19	Day 7	0	0	5	12	0	35,71
20	Day 7	1	0	7	9	0	35,92
21	Day 7						
22	Day 7	1	0	0	6	0,5	35,32
23	Day 7	0	0	6	9	0,5	36
24	Day 7	0	0	7	5	0	34,99

13	Day 14	2	0	0	4	0	35,83
14	Day 14	1	0	4	4	0	35,62
15	Day 14	1	0	9	10	0	34,3
16	Day 14	1	0	9	7	0	35,33
17	Day 14	1	0	4	4	0	35,1
18	Day 14	0	0	2	7	0,5	35,2
19	Day 14	0	0	13	8	0	35,12
20	Day 14	1	0	3	12	0	35,5
21	Day 14						
22	Day 14	0	0	3	12	0,5	35,7
23	Day 14	1	0	5	5	0	35,78
24	Day 14	1	0	2	7	0	33,75
13	Day 21	2	0	0	5	0	35,2
14	Day 21	1	1	3	3	0	34,8
15	Day 21	2	0	3	5	0	33,57
16	Day 21	2	1	9	3	0	32,57
17	Day 21	2	0	4	6	0	33,73
18	Day 21	1	0	4	11	0,5	32,86
19	Day 21	1	0	7	11	0	32,63
20	Day 21	2	0	4	6	0	35,12
21	Day 21	2					
22	Day 21	1	0	7	5	0	33,46
23	Day 21	2	0	4	3	0	34,39
24	Day 21	2	0	3	9	0	33,82
13	Day 28	3					
14	Day 28	2					
15	Day 28	2					
16	Day 28	2					
17	Day 28	2					
18	Day 28	2					
19	Day 28	2					
20	Day 28	2					
21	Day 28						
22	Day 28	2					
23	Day 28	3					
24	Day 28	2					
