# Supplementary material for: A refinement approach in a mouse model of rehabilitation research. Analgesia strategy, reduction approach and infrared thermography in spinal cord injury
Source: PLoS One. 2019 Oct 30;14(10):e0224337. doi: 10.1371/journal.pone.0224337 (PMC6821094; doi:10.1371/journal.pone.0224337)
Supplement: S2 File — (PDF) [file pone.0224337.s002.pdf]

| ID | Day | BMS | Twitching | Grooming | Digging | Ear | Temp30 |
|---|---|---|---|---|---|---|---|
| | | *Score* | *n°event/10 min* | *n°event/10 min* | *n°event/10 min* | *Score* | |
| 25 | Baseline | NA | 0 | 9 | 8 | 0 | 34,94 |
| 26 | Baseline | NA | 0 | 5 | 5 | 0 | 34,28 |
| 27 | Baseline | NA | 0 | 6 | 15 | 0 | 34,5 |
| 28 | Baseline | NA | 0 | 6 | 9 | 0 | 34,67 |
| 29 | Baseline | NA | 0 | 3 | 15 | 0 | 34,5 |
| 30 | Baseline | NA | 0 | 6 | 12 | 0 | 33,62 |
| 37 | Baseline | NA | 0 | 5 | 13 | 0 | 33,28 |
| 38 | Baseline | NA | 0 | 2 | 8 | 0 | 32,68 |
| 39 | Baseline | NA | 0 | 2 | 9 | 0 | 33,42 |
| 42 | Baseline | NA | 0 | 2 | 5 | 0 | 29,88 |
| 47 | Baseline | NA | 0 | 3 | 6 | 0 | 34,38 |
| 48 | Baseline | NA | 0 | 10 | 8 | 0 | 34,7 |
| 31 | Baseline | NA | 0 | 6 | 13 | 0 | 35,38 |
| 32 | Baseline | NA | 0 | 14 | 10 | 0 | 34,37 |
| 33 | Baseline | NA | 0 | 4 | 18 | 0 | 34,12 |
| 34 | Baseline | NA | 0 | 6 | 6 | 0 | 34,07 |
| 35 | Baseline | NA | 0 | 5 | 5 | 0 | 35,29 |
| 36 | Baseline | NA | 0 | 6 | 5 | 0 | 33,83 |
| 40 | Baseline | NA | 0 | 4 | 6 | 0 | 32,06 |
| 41 | Baseline | NA | 0 | 6 | 9 | 0 | 31,84 |
| 43 | Baseline | NA | 0 | 6 | 7 | 0 | 35,1 |
| 44 | Baseline | NA | 0 | 6 | 4 | 0 | 32,84 |
| 45 | Baseline | NA | 0 | 10 | 13 | 0 | 32,78 |
| 46 | Baseline | NA | 0 | 6 | 6 | 0 | 32,6 |
| 25 | Day 0 | 0 | 9 | 0 | 0 | 2 | 35,82 |
| 26 | Day 0 | 0 | 4 | 0 | 0 | 2 | 36,32 |
| 27 | Day 0 | 0 | 9 | 3 | 0 | 2 | 36,98 |
| 28 | Day 0 | 0 | 6 | 0 | 0 | 2 | 34,5 |
| 29 | Day 0 | 0 | 3 | 11 | 0 | 2 | 37,08 |
| 30 | Day 0 | 0 | 18 | 0 | 0 | 1 | 33,96 |
| 37 | Day 0 | 0 | 22 | 8 | 0 | 2 | 32,63 |
| 38 | Day 0 | 0 | 23 | 0 | 0 | 2 | 30,93 |
| 39 | Day 0 | 0 | 5 | 0 | 0 | 2 | 33,08 |
| 42 | Day 0 | 0 | 3 | 1 | 1 | 1.5 | 33,8 |
| 47 | Day 0 | 0 | 0 | 4 | 0 | 1 | 35,66 |
| 48 | Day 0 | 0 | 19 | 0 | 0 | 1.5 | 32,9 |
| 31 | Day 0 | 0 | 2 | 1 | 0 | 1.5 | 35,47 |
| 32 | Day 0 | 0 | 0 | 1 | 0 | 1.5 | 36,07 |
| 33 | Day 0 | 0 | 1 | 0 | 0 | 2 | 34,27 |
| 34 | Day 0 | 0 | 1 | 1 | 0 | 1.5 | 35,9 |
| 35 | Day 0 | 0 | 0 | 4 | 0 | 1.5 | 36,1 |
| 36 | Day 0 | 0 | 5 | 2 | 0 | 1.5 | 35,2 |
| 40 | Day 0 | 0 | 4 | 0 | 0 | 1 | 33,47 |
| 41 | Day 0 | 0 | 1 | 1 | 0 | 0.5 | 33,74 |
| 43 | Day 0 | 0 | 5 | 9 | 0 | 0.5 | 33,86 |
| 44 | Day 0 | 0 | 2 | 1 | 0 | 1.5 | 31,76 |
| 45 | Day 0 | 0 | 1 | 12 | 0 | 1 | 32,72 |
| 46 | Day 0 | 0 | 0 | 0 | 0 | 1.5 | 32,46 |

| | | | | | | | |
|---|---|---|---|---|---|---|---|
| 25 | Day 1 | 0 | 1 | 4 | 0 | 2 | |
| 26 | Day 1 | 0 | 1 | 12 | 2 | 1 | 37,86 |
| 27 | Day 1 | 0 | 0 | 3 | 0 | 2 | 36,62 |
| 28 | Day 1 | 0 | 1 | 8 | 0 | 1.5 | 37,76 |
| 29 | Day 1 | 0 | 0 | 1 | 5 | 2 | 37,78 |
| 30 | Day 1 | 0 | 1 | 0 | 0 | 2 | 36,77 |
| 37 | Day 1 | | | | | | |
| 38 | Day 1 | 0 | 4 | 2 | 0 | 2 | 35,52 |
| 39 | Day 1 | 0 | 1 | 0 | 0 | 2 | 35,32 |
| 42 | Day 1 | 0 | 3 | 3 | 0 | 1 | 36,08 |
| 47 | Day 1 | 0 | 4 | 8 | 5 | 0.5 | 37,84 |
| 48 | Day 1 | 0 | 18 | 0 | 0 | 1.5 | 29,43 |
| 31 | Day 1 | 0 | 0 | 2 | 3 | 0 | 36,02 |
| 32 | Day 1 | 0 | 0 | 1 | 4 | 0.5 | 37,32 |
| 33 | Day 1 | 0 | 0 | 6 | 3 | 0 | 35,79 |
| 34 | Day 1 | 0 | 1 | 6 | 3 | 0.5 | 36,46 |
| 35 | Day 1 | 0 | 0 | 4 | 4 | 0.5 | 37,18 |
| 36 | Day 1 | 0 | 0 | 5 | 2 | 1 | 36,96 |
| 40 | Day 1 | 0 | 3 | 7 | 3 | 0.5 | 36,14 |
| 41 | Day 1 | 0 | 3 | 8 | 1 | 0 | 36,36 |
| 43 | Day 1 | 0 | 2 | 1 | 0 | 0.5 | 36,1 |
| 44 | Day 1 | 0 | 1 | 5 | 0 | 1 | 36,24 |
| 45 | Day 1 | 0 | 0 | 5 | 4 | 1 | 35,96 |
| 46 | Day 1 | 0 | 2 | 9 | 0 | 1 | 35,78 |
| 25 | Day 7 | 1 | 0 | 19 | 6 | 0.5 | 39,58 |
| 26 | Day 7 | 0 | 0 | 10 | 8 | 0.5 | 39,28 |
| 27 | Day 7 | 0 | 0 | 7 | 4 | 0.5 | 39,3 |
| 28 | Day 7 | 1 | 0 | 11 | 3 | 0.5 | 39,72 |
| 29 | Day 7 | 1 | 0 | 4 | 4 | 0 | 40,52 |
| 30 | Day 7 | 0 | 0 | 1 | 12 | 0.5 | 39,77 |
| 37 | Day 7 | | | | | | |
| 38 | Day 7 | 0 | 0 | 5 | 7 | 0 | 36,2 |
| 39 | Day 7 | 1 | 0 | 9 | 8 | 0 | 36,86 |
| 42 | Day 7 | 1 | 0 | 2 | 12 | 0 | 36,1 |
| 47 | Day 7 | 0 | 0 | 3 | 12 | 0.5 | 36,56 |
| 48 | Day 7 | | | | | | |
| 31 | Day 7 | 1 | 0 | 2 | 11 | 0.5 | 39,24 |
| 32 | Day 7 | 0 | 0 | 5 | 9 | 0 | 39,26 |
| 33 | Day 7 | 0 | 0 | 4 | 9 | 0.5 | 38,52 |
| 34 | Day 7 | 1 | 0 | 0 | 9 | 0.5 | 37,98 |
| 35 | Day 7 | 1 | 0 | 4 | 2 | 0.5 | 38,95 |
| 36 | Day 7 | 1 | 0 | 1 | 8 | 1 | 38,05 |
| 40 | Day 7 | 0 | 0 | 12 | 11 | 0 | 36,52 |
| 41 | Day 7 | 1 | 0 | 8 | 12 | 0 | 37,3 |
| 43 | Day 7 | 0 | 0 | 5 | 11 | 0 | 36,98 |
| 44 | Day 7 | 1 | 0 | 14 | 11 | 0 | 36,9 |
| 45 | Day 7 | 0 | 0 | 13 | 11 | 0 | 37,14 |
| 46 | Day 7 | 0 | 0 | 6 | 7 | 0.5 | 36,62 |
| 25 | Day 14 | 2 | 0 | 19 | 1 | 0.5 | 40,37 |
| 26 | Day 14 | 1 | 0 | 10 | 5 | 0.5 | 39,77 |

| | | | | | | | |
|---|---|---|---|---|---|---|---|
| 27 | Day 14 | 1 | 0 | 9 | 2 | 0 | 40,13 |
| 28 | Day 14 | 1 | 0 | 8 | 3 | 0.5 | 40,1 |
| 29 | Day 14 | 1 | 0 | 1 | 15 | 0 | 39,97 |
| 30 | Day 14 | 0 | 0 | 11 | 5 | 1 | 39,98 |
| 37 | Day 14 | | | | | | |
| 38 | Day 14 | 1 | 0 | 9 | 7 | 0 | 33,48 |
| 39 | Day 14 | 1 | 0 | 14 | 4 | 0.5 | 33,68 |
| 42 | Day 14 | 1 | 0 | 5 | 14 | 0.5 | 33,12 |
| 47 | Day 14 | 0 | 0 | 4 | 5 | 0 | 34,73 |
| 48 | Day 14 | | | | | | |
| 31 | Day 14 | 1 | 0 | 6 | 1 | 0.5 | 39,02 |
| 32 | Day 14 | 1 | 0 | 0 | 7 | 0.5 | 39,48 |
| 33 | Day 14 | 1 | 0 | 9 | 4 | 1 | 39,45 |
| 34 | Day 14 | 1 | 0 | 0 | 4 | 0 | 40,3 |
| 35 | Day 14 | 1 | 0 | 3 | 3 | 0 | 38,43 |
| 36 | Day 14 | 1 | 0 | 10 | 2 | 0.5 | 39,94 |
| 40 | Day 14 | 0 | 0 | 7 | 1 | 0 | 34,4 |
| 41 | Day 14 | 1 | 0 | 7 | 8 | 0 | 34,32 |
| 43 | Day 14 | 1 | 0 | 9 | 5 | 0 | 33,44 |
| 44 | Day 14 | 0 | 0 | 4 | 2 | 0 | 33,9 |
| 45 | Day 14 | 1 | 0 | 6 | 6 | 0 | 33,18 |
| 46 | Day 14 | 1 | 0 | 4 | 1 | 0 | 33,88 |
| 25 | Day 21 | 2 | 0 | 12 | 4 | 0.5 | 34,27 |
| 26 | Day 21 | 1 | 0 | 18 | 5 | 0 | 33,75 |
| 27 | Day 21 | 2 | 0 | 10 | 8 | 0.5 | 33,16 |
| 28 | Day 21 | 2 | 0 | 12 | 6 | 0.5 | 32,99 |
| 29 | Day 21 | 2 | 0 | 4 | 7 | 0.5 | 32,09 |
| 30 | Day 21 | 1 | 0 | 9 | 2 | 0.5 | 32,85 |
| 37 | Day 21 | | | | | | |
| 38 | Day 21 | 2 | 0 | 6 | 5 | 0 | 31,62 |
| 39 | Day 21 | 2 | 0 | 9 | 3 | 0.5 | 30,86 |
| 42 | Day 21 | | | | | | |
| 47 | Day 21 | 1 | 0 | 2 | 10 | 0 | 32,26 |
| 48 | Day 21 | | | | | | |
| 31 | Day 21 | 2 | 0 | 2 | 4 | 0 | 32,5 |
| 32 | Day 21 | 1 | 0 | 5 | 2 | 0.5 | 30,39 |
| 33 | Day 21 | 2 | 0 | 6 | 4 | 0 | 30,03 |
| 34 | Day 21 | 2 | 0 | 10 | 1 | 0 | 31,89 |
| 35 | Day 21 | 1 | 0 | 10 | 2 | 0.5 | 31,06 |
| 36 | Day 21 | 1 | 0 | 9 | 1 | 0 | 32,3 |
| 40 | Day 21 | 1 | 0 | 4 | 6 | 0 | 30,5 |
| 41 | Day 21 | 2 | 0 | 4 | 9 | 0 | 31,7 |
| 43 | Day 21 | 2 | 0 | 3 | 3 | 0 | 31,26 |
| 44 | Day 21 | 1 | 0 | 10 | 7 | 0 | 31,78 |
| 45 | Day 21 | 2 | 0 | 4 | 10 | 0 | 30,82 |
| 46 | Day 21 | 2 | 0 | 9 | 5 | 0 | 29,46 |
| 25 | Day 28 | 3 | | | | | |
| 26 | Day 28 | 2 | | | | | |
| 27 | Day 28 | 2 | | | | | |
| 28 | Day 28 | 2 | | | | | |

| | | |
|---|---|---|
| 29 | Day 28 | 2 |
| 30 | Day 28 | 2 |
| 37 | Day 28 | |
| 38 | Day 28 | 2 |
| 39 | Day 28 | 2 |
| 42 | Day 28 | |
| 47 | Day 28 | 2 |
| 48 | Day 28 | |
| 31 | Day 28 | 3 |
| 32 | Day 28 | 2 |
| 33 | Day 28 | 3 |
| 34 | Day 28 | 3 |
| 35 | Day 28 | 2 |
| 36 | Day 28 | 1 |
| 40 | Day 28 | 2 |
| 41 | Day 28 | |
| 43 | Day 28 | 2 |
| 44 | Day 28 | 2 |
| 45 | Day 28 | 3 |
| 46 | Day 28 | 2 |
